# Retraction: Corrective Efficacy of Calcaneal Lengthening Osteotomy for Planovalgus Deformity in Cerebral Palsy Patients

**DOI:** 10.7759/cureus.r167

**Published:** 2025-01-31

**Authors:** Vinod Dubey, Sohilkhan R Pathan, Dhruv Sharma

**Affiliations:** 1 Orthopaedic Surgery, Shree Krishna Hospital and Medical Research Centre, Pramukhswami Medical College, Bhaikaka University, Anand, IND; 2 Clinical Research Services (CRS), Bhanubhai and Madhuben Patel Cardiac Centre, Shree Krishna Hospital and Medical Research Centre, Anand, IND

This article has been retracted by the Editors-in-Chief per the request of the authors due to the following issues:

1. The study was reviewed, but not formally approved by the governing IRB.
2. Prior to publication, consent to use data was not obtained prior to publication from the head of the department nor the institution that owns the data.
3. The co-investigators mentioned in the IRB proposal are not co-authors on the published article and state they have not reviewed the manuscript or the data supporting it. Since the author was a trainee at the institution, all research has to be under supervision and guaranteed by his supervisors. Since this is not the case, the authenticity of the date is in question.

Given these issues, the journal has no choice but to retract this article.

